# Serological evidence of exposure to *Trypanosoma vivax* in cattle from four regions of Colombia assessed by a point-of-care test

**DOI:** 10.1590/S1984-29612026043

**Published:** 2026-10-05

**Authors:** Veronica Camacho, Luiz Ricardo Gonçalves, Marcia Mariza Jusi Merino, Rosangela Zacarias Machado

**Affiliations:** 1 Ceva Animal Health, North Andean Zone, Bogotá, Colombia; 2 Imunodot Diagnóstico Avançado – IMUNODOT, Jaboticabal, SP, Brasil; 3 Universidade Estadual Paulista – UNESP, Faculdade de Ciências Agrárias e Veterinárias – FCAV, Departamento de Patologia, Reprodução e Saúde Única, Vector-Borne Bioagents Laboratory – VBBL, Jaboticabal, SP, Brasil

**Keywords:** Bovine trypanosomosis, cattle production systems, point-of-care test, Tripanosomose bovina, sistemas de produção de gado, teste rápido

## Abstract

*Trypanosoma vivax* is one of the most pathogenic hemoparasites affecting ruminants, causing substantial economic losses in cattle production. Despite its importance, diagnosis remains challenging in several regions, limiting effective disease control. This study aimed to assess exposure to *T. vivax* infection in cattle from four regions of Colombia using a lateral flow point-of-care test (IMUNOTESTE^®^, Imunodot Diagnóstico Avançado) and to analyze its distribution according to different epidemiological variables. A total of 295 cattle of different breeds, ages, and production types were sampled, including animals with clinical suspicion of trypanosomosis and those from areas with previous reports of the disease. Overall, 48.1% of the animals (95% CI: 42.6–54.3%) were seropositive for *T. vivax*. Exposure was significantly associated with geographic region and production system, with the highest seropositivity observed in Santander (60%) and beef herds (57.4%), respectively, whereas no significant association was observed with age group (*p* = 0.179). The most common clinical findings among seropositive animals were cachexia and poor body condition. These results contribute to a better understanding of the epidemiology of *T. vivax* in Colombia and highlight the usefulness of rapid diagnostic tests as effective screening tools for disease surveillance and control.

## Resumo

*Trypanosoma vivax* é um dos hemoparasitos mais patogênicos que acometem ruminantes e causa perdas econômicas expressivas na bovinocultura. Entretanto, o diagnóstico da infecção ainda é desafiador em diversas regiões, limitando a adoção de medidas eficazes para o controle da enfermidade. Este estudo teve como objetivo avaliar a exposição à *T. vivax* em bovinos de quatro regiões da Colômbia por meio de um teste rápido de fluxo lateral (IMUNOTESTE^®^ – Imunodot Diagnóstico Avançado) e analisar sua distribuição em função de diferentes variáveis epidemiológicas. Foram avaliados 295 bovinos de diferentes raças, idades e finalidades produtivas, incluindo animais com suspeita clínica de tripanosomose e provenientes de áreas ou rebanhos com histórico prévio da doença. No total, 48,1% (95% CI: 42.6–54.3%) dos animais foram soropositivos para *T. vivax*. A exposição associou-se significativamente à região e ao sistema de produção, com maior soropositividade em Santander (60%) e em rebanhos de corte (57,4%), respectivamente, enquanto a faixa etária não apresentou associação significativa (*p* = 0,179). Entre os animais soropositivos, os achados clínicos mais frequentes foram caquexia e má condição corporal. Os resultados ampliam o conhecimento sobre a epidemiologia do *T. vivax* na Colômbia e reforçam a utilidade dos testes rápidos como ferramentas de triagem para programas de vigilância e controle.

## Introduction

In cattle, vector-borne diseases such as trypanosomosis represent one of the major causes of economic losses in low-altitude areas, where biting flies of the genera *Tabanus, Stomoxys*, and *Haematobia* are endemic and mechanically transmit *Trypanosoma* parasites during blood feeding (Jaimes-Dueñez et al., 2017; Zapata Salas et al., 2017). However, these flagellates can also be transmitted through infected needles and syringes during the administration of medications (Melo et al., 2022).

In Colombia, the reported prevalence of *T. vivax* ranges from 0.2% to 23% based on molecular methods (García et al., 2014; Jaimes-Dueñez et al., 2018). This parasite is associated with the development of bovine trypanosomosis, which, according to previous reports, has caused losses of up to $5.2 million per year (Chávez-Larrea et al., 2021). Based on the distribution of the vectors, the main regions susceptible to the disease are Magdalena Medio and Piedemonte Llanero, areas where livestock production predominates (Colombia, 2024).

Bovine trypanosomosis, like other parasitic diseases such as babesiosis and anaplasmosis, occurs as periodic epizootics. Similarly, co-infections with *Trypanosoma* spp. and *Anaplasma* spp. or *Babesia* spp. are frequently observed, which may lead to the underdiagnosis of trypanosomosis (Osório et al., 2008).

Affected cattle may remain subclinically infected or develop nonspecific clinical signs characterized by intermittent fever, anemia, abortion, anorexia, decreased milk production, weight loss, and neurological signs such as incoordination, blindness, and muscle tremors (Batista et al., 2007; Jaimes-Dueñez et al., 2021).

In Colombia, the diagnosis of bovine trypanosomosis remains a major challenge for the livestock sector due to the limited availability of affordable diagnostic tests capable of providing rapid and accurate results, which are essential for timely veterinary intervention. Therefore, the objective of this study was to determine the seropositivity to *T. vivax* among cattle from herds located in selected regions of the Colombian low tropics using the IMUNOTESTE^®^ – *Trypanosoma vivax* (Rapid Test) diagnostic kit (Imunodot Diagnóstico Avançado, Jaboticabal, SP, Brazil).

## Material and Methods

### Animals and sampling sites

From July to August 2025, 295 cattle were sampled across four distinct regions of Colombia (Figure 1). The animals were selected from 20 herds ranging in size from 45 to 1,900 animals, representing a total population of 7,481 cattle. A purposive non-probability sampling strategy based on clinical and epidemiological criteria was used. Animals representing different breeds and age groups (< 2 years, 2-5 years, and > 5 years), including both males and females, were selected from beef, breeding, specialized dairy, and dual-purpose farms located in the regions of Caldas, Santander, Caquetá, and Casanare (Table 1). Inclusion criteria included the presence of clinical signs compatible with trypanosomosis, such as poor body condition, anemia, lethargy, abortion, fever, and neurological signs (Osório et al., 2008; Riet-Correa et al., 2025) or animals originating from herds with a previous diagnosis of the disease.

**Figure 1:**
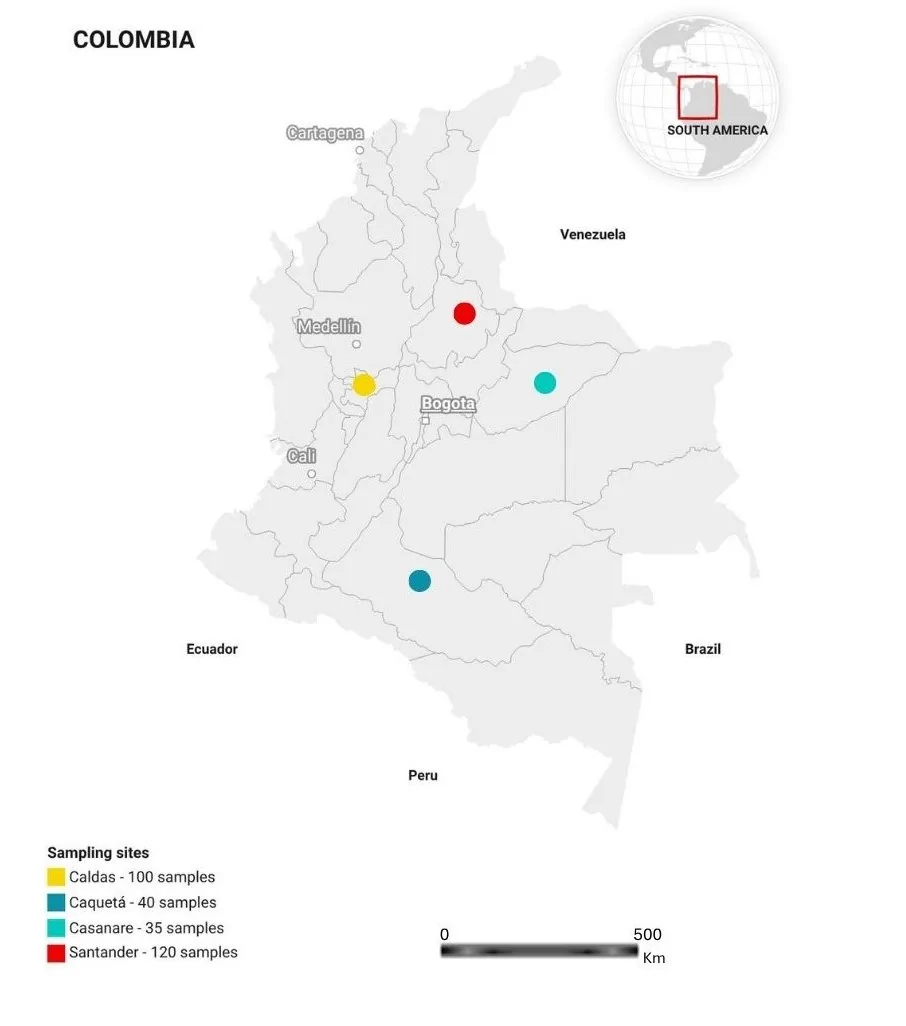
Geographical distribution of the sampling sites in Colombia.

**Table 1:** Number of tests performed by region, production system, breed, sex, and age group.

**Region**	**Animals number**	**Production system**	**Breed**	**Gender**	**Age group**
Santander	120	Beef (120)	Zebu (120)	Male (120)	< 2 years (73)
					2 – 5 years (25)
					> 5 years (22)
Caquetá	40	Dual purpose (40)	Cross breeding (40)	Female (37)	< 2 years (3)
				Male (3)	2 – 5 years (14)
					> 5 years (23)
Caldas	100	Breeding (19)	Norman (15)	Female (99)	2 – 5 years (17)
			Holstein (65)		
			Gyr (1)		> 5 years (83)
		Specialized dairy (81)	Cross breeding (19)	Male (1)	
Casanare	35	Beef (10)	Brahman (25)	Female (25)	< 2 years (3)
		Breeding (15)	Gyr (6)	Male (10)	2 – 5 years (26)
		Dual purpose (10)	Cross breeding (4)		> 5 years (6)

Clinical signs were assessed during examination by a veterinarian from the research team responsible for sample collection. The examination included an assessment of mucous membrane color, body condition, posture, tick infestation, and heart and respiratory rates.

Blood samples were collected from the jugular or caudal vein using tubes containing a clot activator (Vacuplast, code 3269NR). The samples were kept at room temperature until clot formation and then centrifuged at 1,500 × g for 10 min to separate the serum. The serum was transferred to sterile microtubes and stored at −20 °C until serological analysis using the IMUNOTESTE^®^ – *Trypanosoma vivax* (Rapid Test) diagnostic kit (Imunodot Diagnóstico Avançado). According to the manufacturer, the assay is based on a recombinant protein and has demonstrated diagnostic sensitivity and specificity of 95% and 100%, respectively (Imunodot Diagnósticos).

### Serological assay

The IMUNOTESTE^®^ – *Trypanosoma vivax* is based on the principle of lateral flow immunochromatography for the detection of anti-*T. vivax* IgG/IgM antibodies in bovine serum. The assay was performed according to the manufacturer’s instructions (Imunodot, 2021). Briefly, with the aid of a capillary tube provided by the kit, 20 µL of serum was dispensed onto the test device, followed by a drop of buffer solution. The results were read after 15–20 minutes. The results were interpreted visually. A colored line in the control region (C) was required to validate the test. Samples showing a colored line in the test region (T) were considered seropositive for *T. vivax*, while samples without a line in the test region were considered negative.

### Statistical analysis

Data were analyzed using descriptive and inferential statistics. *Trypanosoma vivax* seroprevalence was calculated for each category, together with their respective 95% confidence intervals (95% CI) calculated using the Clopper–Pearson exact method. Associations between disease occurrence and categorical variables (production system, age group, and region) were assessed using Pearson’s chi-square (χ^2^) test or Fisher’s exact test, when appropriate. Statistical significance was defined as p < 0.05. When significant associations were detected, post hoc pairwise comparisons of proportions were performed to identify specific differences between categories and to assign the superscript letters presented in the tables. All statistical analyses were performed using R software.

## Results

Out of the 295 cattle serum samples analyzed, 48.1% (142/295) were seropositive by the rapid test for anti–*T. vivax* antibodies (95% confidence interval: 42.6–54.3%). Among the 20 herds evaluated, 17 (85%) had at least one seropositive animal (Table 2). Santander had the highest seropositivity rate (60%) and differed significantly from Caldas (χ^2^ = 7.14; *p* = 0.007), Casanare (χ^2^ = 7.54; *p* = 0.006) and Caquetá (χ^2^ = 5.05; *p* = 0.024) (*p* < 0.05) (Table 2).

**Table 2:** Number of herds evaluated by region, population per herd, and results of positivity to the IMUNOTESTE rapid test for *T. vivax.*

**Region**	**Number of herds**	**Number of positive herds**	**Population**	**Number of animals**	**Positive herds (%)**	**Positives animals (%)**
Caldas	7	6	725	100	85.7	42^b^ (95% CI: 32.2 – 52.3%)
Caquetá	7	5	881	40	71.4	40^b^ (95% CI: 24.9 – 56.7%)
Casanare	3	3	2525	35	100	34.3^b^ (95% CI: 19.1 – 52.2%)
Santander	3	3	3350	120	100	60^a^ (95% CI: 50.7 – 68.8%)
**TOTAL**	20	17	7481	295	85	48.1 (95% CI: 42.6 – 54.3%

Percentages followed by different superscript letters differ significantly (*p* < 0.05). Pairwise comparisons among regions were performed using Pearson’s chi-square.

Regarding production type, the highest seropositivity rate was observed in beef herds (57%), followed by specialized dairy, dual-purpose, and breeding herds, which showed seropositivity rates of 52.5%, 40%, and 17.6%, respectively (Table 3). Statistical analysis using Pearson’s chi-square (χ^2^) test revealed significant associations between *T. vivax* seroprevalence and production system. Beef and specialized dairy systems showed the highest seroprevalence rates (57.4% and 52.5%, respectively) and differed significantly from the breeding system (17.6%; χ^2^ = 16.92, *p* < 0.001 for beef; χ^2^ = 11.35, *p* = 0.0008 for specialized dairy). In addition, the dual-purpose system (40.0%) showed a significantly higher prevalence than the breeding system (χ^2^ = 4.82, *p* = 0.028) and a significantly lower prevalence than the beef system (χ^2^ = 4.45, *p* = 0.034). No significant differences were observed between beef and specialized dairy systems (χ^2^ = 0.48, *p* = 0.488) (Table 3).

**Table 3:** Seropositivity results for bovine trypanosomosis according to production type and region.

**Production type**	**Region**	**Number of herds**	**Number of animals**	**Number of positives**	**Seropositivity by region (%)**	**Seropositivity by production type (%)**
Beef	Casanare	1	10	2	20	57^a^ (95% CI: 47.9 – 65.6%)
	Santander	3	120	72	60	
Breeding	Caldas	1	20	0	0	17.6^c^ (95% CI: 6.6 – 33.6%)
	Casanare	1	15	6	40	
Dual purpose	Caquetá	7	40	16	40	40^b^ (95% CI: 26.4 – 54.8%)
	Casanare	1	10	4	40	
Specialized dairy	Caldas	6	80	42	52.5	52.5^a,b^ (95% CI: 41.0 – 63.8%)

Prevalence values followed by different superscript letters indicate statistically significant differences (*p* < 0.05). Pairwise comparisons among production systems were performed using Pearson’s χ^2^ test.

Among the breeds evaluated, Zebu crossbreed, and Norman cattle had the highest seropositivity rates (60.8% [73/120] and 60% [9/15]), whereas Gyr (28.5% [2/7]), and crossbred (28.5% [18/63]) cattle had substantially lower seropositivity rates. Additionally, 50.7% (33/65) of Holstein, and 32% of Brahman (8/25) animals tested positive.

Regarding age group, Pearson’s chi-square test showed no significant association between *T. vivax* seroprevalence and age group (χ^2^ = 3.44, *p* = 0.179). The seropositivity was 55.7% (44/79) in animals younger than 2 years, 51.2% (42/82) in animals aged 2–5 years, and 41.8% (56/134) in animals older than 5 years.

The clinical examination performed during sample collection revealed the presence of several clinical signs, some associated with trypanosomosis. Among the clinical signs associated with *T. vivax* infection, pale mucous membranes were observed in 53% (8/15) of seropositive animals, poor body condition in 69% (48/69), cachexia in 70.5% (120/170), and general debility in 100% (42/42). Additionally, seropositivity was highest among animals with three clinical signs. Accordingly, 55.0% (88/160) of the animals with one clinical sign were seropositive, compared with 31.5% (6/19) of those with two clinical signs and 95.4% (42/44) of those with three clinical signs.

## Discussion

The results of this study using the IMUNOTESTE^®^ – *Trypanosoma vivax* diagnostic kit revealed a high seropositivity rate (48.1%) among the evaluated cattle, indicating that bovine trypanosomosis remains widely distributed and potentially underdiagnosed in Colombia. The detection of at least one seropositive animal in 85% of herds suggests widespread exposure and is consistent with previous reports describing the persistent circulation of *T. vivax* in low-altitude areas characterized by favorable ecological conditions for hematophagous vectors (Jaimes-Dueñez et al., 2017; Zapata Salas et al., 2017).

The higher seropositivity rate observed in the Santander department (60.5%) may be partially explained by climatic and production-related factors. This region is characterized by a hot and humid environment and a high density of biting flies, which favor the mechanical transmission of *T. vivax*. Similar associations between environmental conditions, vector abundance, and *T. vivax* prevalence have been reported in endemic regions of Brazil (Cadioli et al., 2012). In addition, previous studies in Colombia have reported a higher risk of infection in extensive beef production systems, where greater exposure to vectors and limited preventive health management are common (Jaimes-Dueñez et al., 2021). However, another study conducted in Colombia reported higher infection rates in animals from intensive production systems (Jaimes-Dueñez et al., 2017). These contrasting findings suggest that *T. vivax* occurrence is not exclusively associated with a specific production system, but rather with regional eco-epidemiological conditions, including vector dynamics, herd management practices, sanitary control measures, and local environmental characteristics.

In the present study, beef production systems showed the highest seropositivity rate (57.3%), which was significantly higher than that observed in breeding systems (17.6%). This finding underscores the role of management practices and production systems in modulating infection risk. Interestingly, in Brazil, several outbreaks have been reported in dairy herds, some of which reported mortality rates of up to 50% (Andrade et al., 2019). In these outbreaks, the introduction of new animals and the reuse of contaminated needles and syringes were identified as the main factors associated with *T. vivax* transmission (Riet-Correa et al., 2025). However, it is important to highlight that in the present study, specialized dairy and dual-purpose systems also showed considerable seroprevalence, and the lower frequency observed in breeding herds should be interpreted with caution, given the limited number of herds evaluated in this category.

Breed-related differences in seropositivity were observed, with Zebu crossbreed and Normande cattle showing the highest rates (60.5% and 60%, respectively). These findings may reflect unequal distribution of samples among breeds as well as possible physiological or immunological differences previously described (Mattioli et al., 2000). Zebu breeds, despite their relative resistance to certain hemoparasitic infections, have been reported to harbor chronic *T. vivax* infections and may act as reservoirs within herds (Batista et al., 2007). Conversely, the high seropositivity observed in European breeds such as Holstein and Normande suggests increased clinical susceptibility and a higher potential for productive losses, particularly in dairy systems.

No statistically significant differences were observed among age groups, suggesting relatively uniform exposure to *T. vivax* across different life stages in the studied regions. These findings contrast with reports from Uganda and Colombia, where higher prevalence in adult cattle compared to calves was attributed to prolonged cumulative vector exposure, the waning of maternal immunity, and the preferential attraction of vectors to larger animals (Angwech et al., 2015; Jaimes-Dueñez et al., 2021).

The most frequently observed clinical signs among seropositive animals - cachexia, anemia, and poor body condition - are consistent with the pathophysiology of trypanosomosis, which involves hemolytic processes and immunosuppression (Batista et al., 2007). Moreover, the predominance of multiple clinical signs among seropositive animals highlights the importance of clinical observation in the field. However, the nonspecific nature of these manifestations reinforces the need for laboratory confirmation, as similar signs may be associated with other infectious diseases (not evaluated in this study) or nutritional conditions (Riet-Correa et al., 2025).

The IMUNOTESTE^®^ – *T. vivax* demonstrated promising performance as a rapid and easily interpretable screening tool under field conditions. Its reliability is supported by previous studies showing good agreement between ELISA and the rapid test (Serra et al., 2024). Although indirect tests require cautious interpretation, particularly in endemic settings, the use of point-of-care tests represents an important practical advance given the logistical and technical limitations associated with conventional diagnostic methods. The availability of accessible screening tools may substantially improve early detection and support timely veterinary decision-making.

It is important to highlight that the rapid test used in this study detects both IgG and IgM antibodies, and a positive result does not necessarily indicate active infection, as antibodies may remain detectable even after parasite clearance. Accordingly, some studies using ELISA have demonstrated the persistence of antibodies against *T. vivax* even after treatment with isometamidium hydrochloride (1 mg/Kg) (Castilho Neto et al., 2021). Therefore, the rapid test should be considered primarily as a screening tool, and its results interpreted in conjunction with clinical findings, epidemiological context, and, when necessary, complementary laboratory methods to support a more accurate diagnosis (Fidelis et al., 2019).

The sampling strategy, which included a predominance of animals from areas or herds with a previous history of trypanosomosis or animals presenting clinical signs compatible with the disease, may have introduced selection bias and resulted in an overestimation of seroprevalence. This represents an important limitation of the current study. Additionally, unequal sample sizes among regions, breeds, and production systems limit the generalizability of the findings. Nevertheless, the data provide a valuable overview of the epidemiological situation of bovine trypanosomosis in Colombia.

## Conclusion

In conclusion, this study revealed a high exposure rate to *T. vivax* among cattle with clinical suspicion of trypanosomosis or from herds with a previous history of the disease, confirming that bovine trypanosomosis remains an important animal health and economic challenge for Colombian livestock production. Our results showed that *T. vivax* exposure was associated with geographic region and production system, with the highest seropositivity observed in Santander (60%) and beef herds (57.4%), respectively, but not with age group (*p* = 0.179). The use of rapid diagnostic tests as screening tools proved valuable and may substantially contribute to epidemiological surveillance programs. The high seroprevalence observed across different regions and production systems underscores the need for enhanced surveillance and control programs to mitigate the impact of *T. vivax* on cattle production in Colombia.

## Data Availability

Data are contained within the article.
